# Diminished warming tolerance and plasticity in low-latitude populations of a marine gastropod

**DOI:** 10.1093/conphys/coab039

**Published:** 2021-06-11

**Authors:** Andrew R Villeneuve, Lisa M Komoroske, Brian S Cheng

**Affiliations:** 1 Department of Environmental Conservation, University of Massachusetts Amherst, Amherst, MA 01003, USA; 2 Gloucester Marine Station, University of Massachusetts Amherst, Gloucester, MA 01930, USA

## Abstract

Models of species response to climate change often assume that physiological traits are invariant across populations. Neglecting potential intraspecific variation may overlook the possibility that some populations are more resilient or susceptible than others, creating inaccurate predictions of climate impacts. In addition, phenotypic plasticity can contribute to trait variation and may mediate sensitivity to climate. Quantifying such forms of intraspecific variation can improve our understanding of how climate can affect ecologically important species, such as invasive predators. Here, we quantified thermal performance (tolerance, acclimation capacity, developmental traits) across seven populations of the predatory marine snail (*Urosalpinx cinerea*) from native Atlantic and non-native Pacific coast populations in the USA. Using common garden experiments, we assessed the effects of source population and developmental acclimation on thermal tolerance and developmental traits of F1 snails. We then estimated climate sensitivity by calculating warming tolerance (thermal tolerance − habitat temperature), using field environmental data. We report that low-latitude populations had greater thermal tolerance than their high latitude counterparts. However, these same low-latitude populations exhibited decreased thermal tolerance when exposed to environmentally realistic higher acclimation temperatures. Low-latitude native populations had the greatest climate sensitivity (habitat temperatures near thermal limits). In contrast, invasive Pacific snails had the lowest climate sensitivity, suggesting that these populations are likely to persist and drive negative impacts on native biodiversity. Developmental rate significantly increased in embryos sourced from populations with greater habitat temperature but had variable effects on clutch size and hatching success. Thus, warming can produce widely divergent responses within the same species, resulting in enhanced impacts in the non-native range and extirpation in the native range. Broadly, our results highlight how intraspecific variation can alter management decisions, as this may clarify whether management efforts should be focused on many or only a few populations.

## Teaser

The physiology of populations within species can determine sensitivity to climate change. Experiments on marine snails indicate that southern populations are most susceptible to warming because they have minimal physiological flexibility and are most threatened by extreme heat events. Management of this species depends on the specific population in question.

## Data Accessibility

R scripts and data used in the analyses for this manuscript can be found at: https://github.com/villesci/Uro (Villeneuve *et al.*, 2021).

## Introduction

Understanding the sensitivity of species to climate change is a primary aim of global change ecology (Calosi *et al.*, 2008; Williams *et al.*, 2008; Bennett *et al.*, 2019). Ecological forecasts are a suite of modelling tools that can aid conservation practitioners in determining species sensitivity to climate change by correlating occupied distribution environments or known physiological limits with predictions of future climate scenarios (Pearson and Dawson, 2003; Helmuth, 2009; Chown *et al.*, 2010; Cacciapaglia and van Woesik, 2018). In a conservation and management context, ecological forecasts can be used to identify species at risk and prioritize efforts and management actions on species and ecosystems of concern (Payne *et al.*, 2017; Tulloch *et al.*, 2020). However, these models often use physiological measures from a single population to infer the capacity of a species to respond to environmental change (Pearman *et al.*, 2010; D’Amen *et al.*, 2013; Valladares *et al.*, 2014; Lecocq *et al.*, 2019) and implicitly assume that physiological niches are homogenous across populations within a species (Peterson, 1999, 2011; Bennett *et al.*, 2019). However, populations within species often exhibit physiological variation that reflects heterogeneity in environmental conditions and potential local adaptation (Moran *et al.*, 2016; Peterson *et al.*, 2019). Ignoring the potential for such locally adapted variation greatly risks under- or over-estimating species sensitivity to climate change (Pearman *et al.*, 2010; Valladares *et al.*, 2014; Cacciapaglia and van Woesik, 2018). For example, populations of widely distributed species can differ in thermal tolerance by up to 1.5°C–3.8°C (e.g. Fangue *et al.*, 2006; Pereira *et al.*, 2017). In contrast, thermal tolerance may be invariant across a species range, a pattern that is described as niche conservatism (Lee and Boulding, 2010; Pearman *et al.*, 2010; Gaitán-Espitia *et al.*, 2017). If populations are niche conserved, then modelling a species as a single unit is appropriate. However, the management implications of assuming niche conservatism or local adaptation can be starkly divergent; when modelled as having homogenous physiology throughout its range, a *Porites* coral species was expected to increase its range by 5%–6% by 2100, whereas when modelled as 5 distinct populations the range was forecasted to decrease by 50% (Cacciapaglia and van Woesik, 2018). Thus, intraspecific variation in thermal performance may be crucial to understanding species sensitivity to climate change, but our understanding of mechanisms underlying such variation remains incomplete.

Climate sensitivity may also be mediated by phenotypic plasticity. Acclimation is one form of plasticity that is defined as within generational phenotypic change in response to an altered environmental change and may allow an organism to rapidly adjust physiology to changing environmental conditions (Seebacher *et al.*, 2012; Beaman *et al.*, 2016). For example, higher acclimation temperatures tend to increase thermal tolerance, primarily due to coordinated molecular adjustments such as increased heat shock protein expression to maintain or regain homeostasis (Hofmann, 1999; Basu *et al.*, 2002; Guy *et al.*, 2008). The majority of these studies examine plasticity within a focal life stage (Marshall, 2008; Moore and Martin, 2019). However, organismal life stages do not act as ‘firewalls’ past which the effects of thermal challenge cannot penetrate (Marshall and Morgan, 2011). The effects of marine climate change will impact all life stages of marine organisms and exposure to thermal stress in one life stage can result in latent or carry-over effects to future life stages (Pechenik, 2006; Hettinger *et al.*, 2012). Developmental acclimation should increase adult thermal tolerance to a point, beyond which we expect a reduction in adult tolerance when acclimation temperature exceeds the thermal optima of organismal performance (Overgaard *et al.*, 2011; Scharf *et al.*, 2015; Truebano *et al.*, 2018). Therefore, identifying the potential effects of developmental acclimation are critical to understanding actual organismal reactions in later life stages.

Even though plastic trait expression is often linked to environmental exposure, the extent of plasticity capacity itself can be adapted to local conditions (De Jong, 2005; Valladares *et al.*, 2014). Under the latitudinal variability hypothesis, which predicts how thermal phenotypic plasticity might vary between latitudinally separate populations, high-latitude but non-polar populations should have higher plasticity in response to seasonally variable temperatures (Bozinovic *et al.*, 2011; Gunderson and Stillman, 2015; Barria and Bacigalupe, 2017). In contrast, tropical and polar species that experience minimal seasonality are expected to have lower plasticity in response to limited environmental fluctuations (Tewksbury *et al.*, 2008; Overgaard *et al.*, 2011; Peck *et al.*, 2014). It has also been suggested that lower plasticity in warm adapted populations may reflect a trade-off between plasticity and greater overall tolerance (trade-off hypothesis; Stillman, 2003; Magozzi and Calosi, 2015; Sasaki and Dam, 2019; van Heerwaarden and Kellermann, 2020). Plasticity can buffer species’ susceptibility to warming temperatures, and thus it is important to quantify this trait in order to fully assess warming sensitivity (Palumbi *et al.*, 2014). However, among species variation in plasticity means warming sensitivity will also vary, requiring study on a species-by-species basis to accurately understand warming sensitivity (Seebacher *et al.*, 2012). Considering the role of plasticity, in addition to potential local adaptation, are critical to determining organismal susceptibility to thermal stress (Valladares *et al.*, 2014).

**Figure 1:**
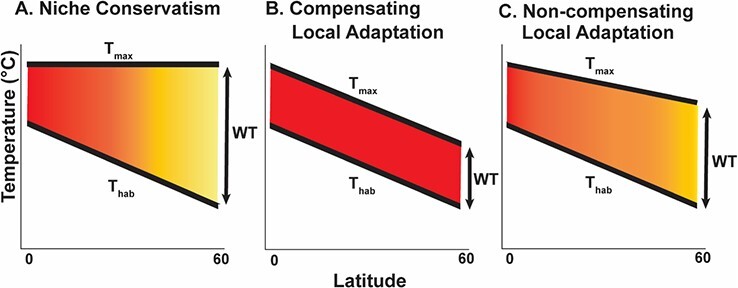
Conceptual diagram of how thermal tolerance (T_max_) and habitat temperature (T_hab_) interact under niche-conserved thermal tolerance (**A**), ‘compensating’ locally adapted thermal tolerance (**B**) and ‘non-compensating’ local adaptation (**C**) to result in differing expectations of WT with latitude; colour shading refers to WT magnitude, with yellow indicating high WT and red indicating low WT values.

Understanding geographic variation in thermal performance is central to identifying which populations may be at the greatest risk of extinction caused by climate change. Both across species and populations, evidence suggests that upper thermal tolerances increase with decreasing latitude (e.g. Stillman and Somero, 2000; Sgrò *et al.*, 2010; Zippay and Hofmann, 2010; Sunday *et al.*, 2011; Pereira *et al.*, 2017; Jensen *et al.*, 2019; Sasaki and Dam, 2019). However, quantifying thermal tolerance alone does not reveal climate sensitivity, as it does not factor in the ‘environmental distance’ between thermal tolerance and the *in situ* temperature regime. It is therefore necessary to integrate habitat temperature with organismal tolerance. An organism’s ‘warming tolerance’ (WT) quantifies this buffer by calculating the difference between thermal tolerance and habitat temperature (e.g. mean annual temperature; Deutsch *et al.*, 2008). In the absence of rapid thermal adaptation, populations at greatest risk of warming are those with diminished WT (Deutsch *et al.*, 2008; Bennett *et al.*, 2019). In populations with invariant thermal limits (niche conservatism), WT may be greater at high latitudes, as the difference between habitat temperature and the conserved thermal tolerance will be large (Fig. 1A; Bennett *et al.*, 2019). In contrast, low-latitude populations would be most sensitive because of the small difference between habitat temperature and thermal tolerance, assuming habitat temperatures decrease more or less linearly from the equator to the poles (Tewksbury *et al.*, 2008; Diamond *et al.*, 2012; Bennett *et al.*, 2019; Pinsky *et al.*, 2019). However, if thermal tolerance varies across populations (‘compensating’ local adaptation), WT may actually be similar across populations, suggesting sensitivity across the entire species range (if WT is low) or resilience to changing temperatures (if WT is high; Fig. 1B; Bennett *et al.*, 2019). Finally, local adaptation in thermal tolerance may exist, but may not track perfectly with habitat temperature (‘non-compensating’ local adaptation), resulting in greater sensitivity to climate warming in populations with greater thermal exposure (Fig. 1C). Thus, integrating intraspecific measures of physiological performance with environmental data is a promising approach that can clarify population sensitivity to climate change. For conservation stakeholders, this integration can better inform whether management needs to be focused on a few sensitive populations, many populations throughout a species range or none.

In a management and conservation context, knowledge of physiological performance can also clarify our understanding of impacts of invasive species under climate change (Zerebecki and Sorte, 2011; Sorte *et al.*, 2013; Lennox *et al.*, 2015). Greater thermal tolerance breadths and plasticity are traits that can contribute to the success of invasive species, particularly in the face of climate change (Chown *et al.*, 2007; Slabber *et al.*, 2007; Sorte *et al.*, 2010; Zerebecki and Sorte, 2011; Seebacher *et al.*, 2012; Kelley, 2014). These adaptations may allow invasive species to survive challenging transport conditions and to rapidly colonize habitats with thermal conditions that differ from their native range (Diez *et al.*, 2012). These same traits are also predicted to confer climate resilience to invasive species as habitats experience elevated and increasingly variable temperatures (Dukes and Mooney, 1999; Stachowicz *et al.*, 2002; Diez *et al.*, 2012; Sorte *et al.*, 2013). Forecasting the impacts of invasive species under climate warming may be informed by knowledge of thermal physiology in both the native and invasive ranges because adaptation in the native range provides the standing genetic material that founds invasive populations. For invasive populations, thermal tolerance and plasticity may be locally adapted to novel environments, even those that are warmer or colder than their native range environment (Beaumont *et al.*, 2009; Griffith *et al.*, 2014; Tepolt and Somero, 2014; Wesselmann *et al.*, 2020). Altogether, there exists a range of possible climate sensitivities of invasive populations that may not be accurately described by native range thermal physiology. Neglecting the potential for novel trait performance in invasive populations can decrease the accuracy of ecological forecasts to climate change that are solely based on the native range (Broennimann *et al.*, 2007; Fitzpatrick *et al.*, 2007; Loo *et al.*, 2007; Beaumont *et al.*, 2009). Thus, evaluating the range of thermal physiology across native and invasive populations of single species can shed light on the range of current adaptations within a species and thus clarify the extent of current sensitivity, as well as the potential for future evolutionary adaptation to climate change (Beaumont *et al.*, 2009; Henkel *et al.*, 2009; Hill *et al.*, 2013; Wesselmann *et al.*, 2020).

To address the roles of local adaptation and plasticity in determining thermal sensitivities across native and invasive ranges, we quantified intraspecific variation in thermal performance of invasive and native populations of an ecologically important predatory marine snail (Atlantic oyster drill, *Urosalpinx cinerea*). We used split-brood common garden experiments to assess thermal performance of laboratory-reared F1 juveniles sourced from native and invasive populations across a latitudinal gradient on the Atlantic (32.7°–43.1° N) and Pacific (38.1°–40.8° N) coasts of the USA, respectively. Our specific objectives were as follows: (i) to determine if variation in thermal tolerance and developmental traits occurs among native and invasive populations, (ii) to quantify plasticity in thermal tolerance and developmental traits by manipulating temperature during embryonic incubation and (iii) to estimate climate sensitivity of each population using WT (Deutsch *et al.*, 2008). We hypothesized that (i) thermal tolerance would increase with environmental temperature, thereby suggesting local adaptation; (ii) elevated acclimation temperature during development would result in greater juvenile thermal tolerance; and (iii) plasticity would be highest in cold origin populations. Because latitude itself is not a perfect predictor of the actual environmental temperatures experienced by populations, particularly across coastal latitudinal gradients, we also evaluated the correlation between a suite of environmental metrics (e.g. maximum and mean temperature) and thermal and WT (Helmuth, 2009; Kuo and Sanford, 2009). Our broader goal was to quantify intraspecific thermal performance across a species’ native and invasive ranges to determine what populations are likely most sensitive to climate warming, and therefore identify which populations of *Urosalpinx* are likely to persist in the long term without management intervention.

## Methods

### Species selection

We used the snail *U. cinerea* (hereafter *Urosalpinx*) as our focal species because of its limited dispersal that drives a high potential for local adaptation, its wide range across latitude and thermal regimes and its tractability in the egg and juvenile life stages (Cheng *et al.*, 2017). *Urosalpinx* undergoes direct development, laying benthic egg cases that each contain 4–16 embryos that develop for 26–56 days after which they emerge as hatchlings (Carriker, 1955). Because of this direct development, dispersal and gene flow are likely limited among populations, suggesting a high potential for local adaptation (Kawecki and Ebert, 2004). Further, we sampled populations from both the invaded and native ranges of *Urosalpinx* with the goal of understanding if trait performance differs between invaded and native populations under different thermal regimes (Zerebecki and Sorte, 2011). *Urosalpinx* is native on the Atlantic coast of North America from south Florida to Massachusetts and cryptogenic (of unknown origin) north to Nova Scotia (Fofonoff *et al.*, 2020). In the late 1800s, *Urosalpinx* was introduced to multiple locations on the Pacific coast of North America, ranging from San Francisco Bay north to Puget Sound, via importation of Eastern oysters (*Crassostrea virginica*; Carriker, 1955; Fofonoff *et al.*, 2020). The high biomass (1.7 million kg) and diverse origins of oysters transported to these Pacific sites (Hoos *et al.*, 2010) indicate initial *Urosalpinx* populations were likely large, suggesting limited founder effects. In the invasive range, *Urosalpinx* can virtually eliminate native oysters and other native species via predation (Carriker, 1955; Kimbro *et al.*, 2009; Cheng and Grosholz, 2016).

### Broodstock collection

**Figure 2:**
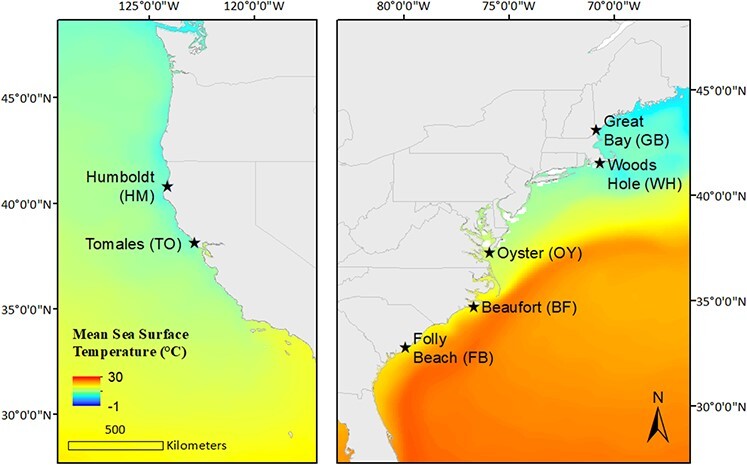
*Urosalpinx cinerea* collection sites on the Atlantic and Pacific seaboards of the USA; mean sea surface temperature (SST) is an annual composite of 2018 5 km data (data source: NOAA/NESDIS Geo-Polar, Maturi *et al.*, 2017; annual SST composite data from NOAA Coral Reef Watch 2018 v3.1).

We examined physiological performance of F1 offspring in order to ensure a common garden environment for the entire embryonic and juvenile life phases. This approach does not fully account for the possibility of maternal or transgenerational effects but is a reasonable starting point for assessing intraspecific patterns of thermal performance. To produce F1 offspring for experimentation, we collected broodstock adult *Urosalpinx* from seven sites: five from the Atlantic and two from the Pacific that encompassed a wide range of their latitudinal distribution (Fig. 2). All collections were conducted from 15 March to 9 June 2019. We chose collection sites to be within 15 km of *in situ* environmental data loggers (e.g. National Data Buoy Center, National Estuarine Reserve System, NOAA Ocean Observing System; Table S1). At each site, we hand collected at least 30 adult male and female oyster drills in the extreme low intertidal and subtidal zones from both natural and artificial substrate, including oyster reefs, pier pilings and boulders, within a 30-meter radius. We then transported snails in aerated coolers of seawater from collection sites, kept cool with ice packs and separated by population. Water conditions within the coolers were monitored to maintain 100% dissolved oxygen saturation and temperature within 4°C of collection temperature. Samples from Humboldt Bay and Tomales Bay (Pacific populations) were collected in a similar fashion except that they were overnight mailed in plastic bags with saltwater-moistened paper towels but without seawater. Snails were kept cool with ice packs and upon arrival were immediately placed in holding tanks separated by population at the University of Massachusetts, Amherst. No mortalities occurred as a result of collection or shipping.

We maintained *Urosalpinx* populations in a recirculating seawater system at 12°C (salinity 30 PSU) until they were needed for experimentation and as other populations were collected. Populations were kept separate in plastic aquaria with aeration. We fed broodstock *Urosalpinx* with blue mussels (*Mytilus edulis*), acorn barnacles (*Semibalanus balanoides*) and eastern oyster flesh (*C. virginica*) *ad libitum*. To induce egg case laying, we raised the system water temperature by 1°C/day until 20°C was reached and then moved all broodstock to an identical recirculating seawater system at the Gloucester Marine Station (UMass Amherst). We performed daily water changes on the broodstock recirculating system using ambient coastal seawater maintained at 20°C. We also monitored ammonia levels (API Mars Fishcare, Inc., Chalfont, PA) to ensure levels stayed below 0.25 ppm. Ammonia varied between 0 and 0.25 ppm with 1 spike to 0.5 ppm caused by overfeeding, remedied with daily water changes.

### Egg case collection and developmental acclimation

Our primary goal was to quantify thermal tolerance and plasticity (measured as developmental acclimation capacity at 20°C and 24°C) across populations. We selected 20°C to enable comparison with prior work on *Urosalpinx* (Cheng et al. 2017) and chose 24°C to represent a warmer temperature that *Urosalpinx* likely already experiences during summer and is below a previously recorded juvenile thermal optima (26.5°C; Cheng *et al.*, 2017). Thus, we hypothesized that an increase in acclimation temperature from 20°C to 24°C would result in an increase in thermal tolerance. We performed daily inspections for egg cases from 5 to 31 July 2019 and collected a total of 122 egg cases. Mothers typically laid eggs in clusters of 5–8 cases. In cases where a mother was discovered laying the egg cluster, we affixed a plastic numbered tag to the mother with cyanoacrylate glue to track the identity of egg-laying mothers. We tracked mother identity, as well as unique clutches (group of egg cases found together), to differentiate egg cases laid by different individual mothers. Because some eggs were laid by unidentified mothers (*n* = 21), we used unique clutches to control for maternal effects.

We collected eggs the day they were laid and incubated them using two methods to facilitate collection of different data types. For development time, we placed single eggs into plastic tea strainers (Tops Permabrew, Darien, CT) that were divided in half with nylon fabric. Each tea strainer therefore held two eggs from a single egg cluster and allowed us to track time to hatching of individual egg cases. For thermal tolerance, the remaining eggs were housed in undivided tea strainers separated by population (20–30 egg cases per strainer) until hatchling emergence. Both types of strainers were submerged in seawater maintained at 20°C or 24°C (salinity, 30 PSU), which served as our developmental acclimation for the egg stage. In each aquarium, we monitored temperature at least twice daily; temperature within the aquaria never varied by more than ±0.4°C for the duration of egg development.

Immediately after hatching, we combined F1 snails from different mothers and of the same population and acclimation temperature into strainers and fed F1 snails *C. virginica* oyster spat *ad libitum* (3 mm shell diameter; Muscongus Bay Aquaculture, Bremen, Maine). F1 snails were housed in strainers between 8 and 16 days submerged within a tank (39 l) maintained at 20°C or 24°C before they were placed in the thermal tolerance experiment, and thus acclimation extended post-hatch. The mass of juvenile snails as recorded immediately before the thermal tolerance experiment was not significantly different between both acclimation temperature treatments (Generalized Linear Model (GLM), F_1,649_ = 2.90, *P* = 0.0892). The egg cases acclimated at 24°C from Great Bay, NH, USA, and Woods Hole, MA, USA, did not produce enough juveniles to enter in a heat bar trial, and so were not included in our analysis.

### Thermal tolerance

We quantified thermal tolerance and developmental acclimation across populations using LT_50_ methodology with an aluminium heat bar (Kuo and Sanford, 2009; Cheng *et al.*, 2017). The heat bar was drilled to accommodate 5-ml centrifuge tubes that can house individual snails that are then exposed to a gradient of temperatures along the length of the heat bar. This heat bar was constructed with a solid aluminium block similar to Kuo and Sanford (2009), but heat was applied with a silicone heating element (Omega SRFGA-406/2-P 60 watt, Omega Engineering, Norwalk, CT, USA) and adjusted with a proportional integral derivative (PID) controller (ITC-100, Inkbird, Shenzhen, PRC). Cooling was maintained by circulating 3°C–5°C water through the opposing end of the heat bar. Although *Urosalpinx* experiences aerial and aquatic thermal stress, this species is commonly found in both subtidal and low-intertidal habitats with limited aerial exposure (Carriker, 1955; Cheng and Grosholz, 2016; Cheng et al., 2017). Thus, we chose to quantify thermal tolerance in water to avoid the confounding effect of aerial desiccation (Stillman and Somero, 2000).

In heat bar trials, individual snails were placed in 5-ml centrifuge tubes filled with 5 ml of aerated seawater at the same acclimation temperature the snail experienced during development. We inserted a 2 × 2-cm, 200-μm nitex mesh square into the tube using a plastic collar so that ~0.5 ml of the tube’s water was above the mesh. This prevented the snail from crawling out of the water, ensured free exchange of oxygen with the water in the tube and enabled us to record water temperature without disturbing the snail. We randomly assigned one of the three possible row positions along the heat bar, so that each population was represented in a column but in a random row. Thus, we tested up to three different population-acclimation treatments (each of which was defined as a ‘trial’) at a time on the heat bar array (Fig. S1). Each heat bar ‘run’ was defined as a ramping of 3 trials in the heat bar with 18–30 snails from 3 populations and a single acclimation temperature. We quantified wet weight of each live snail (Ohaus Pioneer PX Scale, Ohaus Corporation, Parsippany, NJ, USA) prior to the run to account for age and size effects, as age and age-linked size can affect thermal tolerance (Nyamukondiwa and Terblanche, 2009; Truebano *et al.*, 2018). However, there was little evidence that age (as measured by centered and scaled body mass) predicted survivorship (Table S2). Therefore, we removed body mass as a predictor from our models. The shell length of these juvenile snails ranged from 1–2 mm.

We used the PID controller to control the temperature ramp along the heat bar, increasing the controller setpoint by 5°C every 30 minutes in steps from 25°C to 60°C for a total period of 4 hours. In the final hour, we held the heat bar at 60°C, so each snail was exposed to a heat ramp lasting 5 hours (Table S3, Fig. S2). We measured the temperature in each column every hour using a thermocouple. After the heat ramp, we removed the centrifuge tubes from the heat bar and allowed them to recover in aerated seawater at the appropriate acclimation temperature (20°C or 24°C) overnight. After the recovery period, we evaluated snails for mortality using a stereomicroscope and a probe classifying snails that did not retract their foot upon stimulus as dead and those that reacted as alive (Cheng *et al.*, 2017). In total, we conducted 22 independent heat bar trials (20°C, *n* = 14; 24°C, *n* = 8) for 7 populations using a total of 652 juvenile snails (Table S4). Individual snail sample sizes between acclimations were uneven, with 418 at 20°C and 234 at 24°C, due to egg case availability.

### Developmental metrics

In addition to thermal tolerance, we quantified the effects of temperature on development across populations by measuring the following: (i) hatching success, (ii) clutch size and (iii) developmental rate. To assess embryo hatching success over the incubation period, we counted the number of successfully hatched snails and compared this to the number of unsuccessful embryos using a microscope (Leica S9i, Leica Microsystems, Inc, Buffalo Grove, IL, USA). We also counted the number of initial embryos per egg case to evaluate clutch size. To measure developmental rate of embryos within egg cases, we noted the lay date of each case within 2 days of laying and checked egg cases daily for hatching. We classified an entire egg case as hatched when the first hatchling snail emerged from the opening at the top of each egg case, allowing hatchlings to crawl freely out of the egg case.

### Environmental metrics

**Figure 3:**
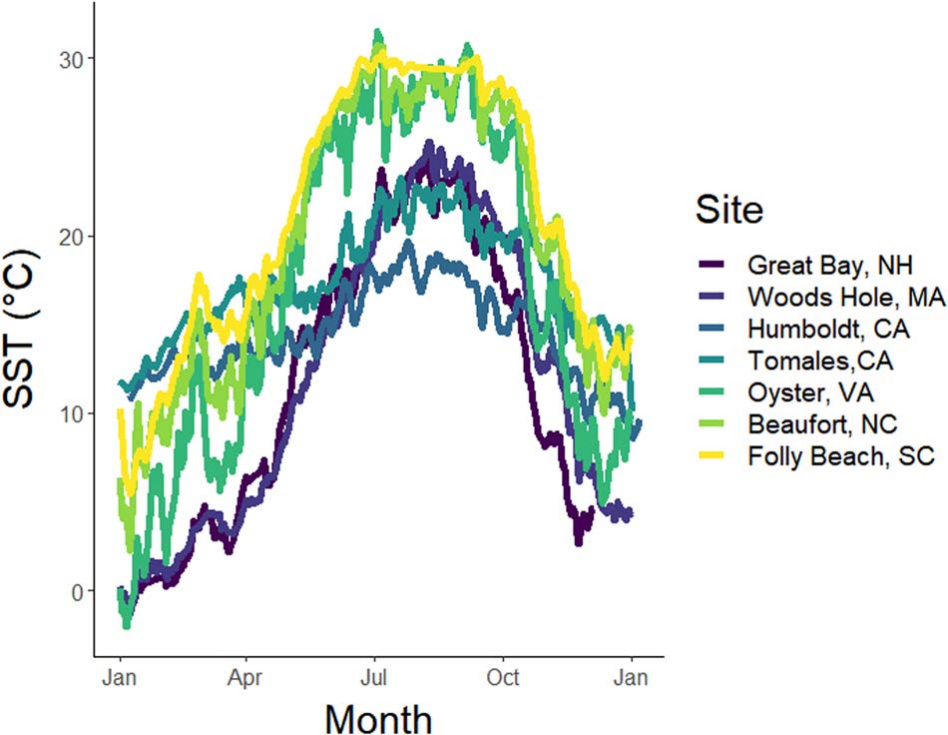
SST from sources near broodstock collection sites, with each time series represents 1 year of data from 1 January to 31 December 2018 (except for Pacific sites, where data ranged from 1 January to 31 December 2015) for comparison of thermal regime across populations; lines represent the daily mean temperature at each site; sites are presented in order of annual mean temperature; see Table S1 for source list and sampling dates.

While latitude is a commonly used metric of the types of environmental conditions experienced by a population (e.g. Sunday *et al.*, 2014), we chose to evaluate multiple site level environmental temperature metrics as potential predictors of thermal tolerance and developmental traits because latitude may not be an accurate predictor of local scale temperatures experienced by organisms (Kuo and Sanford, 2009). Moreover, while latitude can be a useful predictor that is correlated with environmental conditions, habitat temperatures can differ at the same latitude based on ocean (Pacific vs. Atlantic) and local (inner estuary vs. outer estuary) conditions, and is thus another potential direct driver of environmentally adapted traits (Kuo and Sanford, 2009; Baumann and Conover, 2011; Sunday *et al.*, 2011). Thus, we extracted a series of environmental temperature predictors with the goal of understanding what aspect of habitat temperature (e.g. mean vs. maximum temperature) best predicted patterns in thermal tolerance. From these temperature data, we calculated 5 environmental predictors: (i) mean annual temperature, (ii) summer mean temperature, (iii) upper 25th percentile of the summer period, (iv) the upper 10th percentile of the summer period and (v) the maximum summer temperature (Table S5). We used each environmental predictor by itself in each model to evaluate which predictor best explained trait performance patterns using model selection, including a null model. We selected site temperature data based on the completeness of the record in 2018, the proximity of the temperature data to the collection site (no more than 15 km; Table S1) and from locations representative of collection sites (e.g. environmental data was collected from buoys in tidal creeks if the collection site was in a tidal creek). When available, we selected only continuous 2018 temperature records, but the two data sources from the Pacific only had continuous data from 2015 (Table S1; Fig. 3). Summer was classified as between 1 June and 30 September.

### Statistical analysis

#### Thermal tolerance

**Figure 4:**
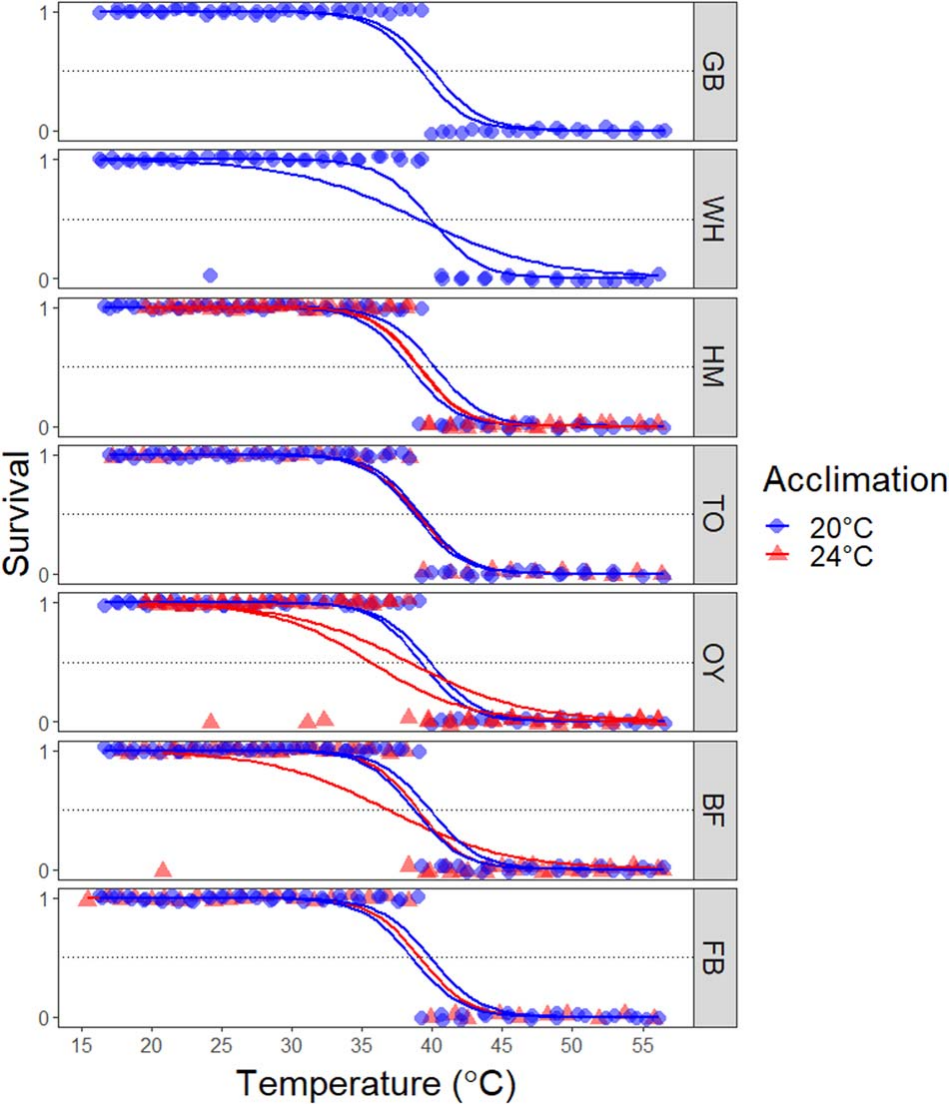
Survivorship of *Urosalpinx* hatchlings (survival, 1; mortality, 0) as a function of final temperature within the heat bar array, separated by acclimation temperature; model estimates represent independent heat bar trials and dotted line represents the threshold for calculating LT_50_. Populations are ordered by ascending mean temperature within the native and invasive (HM and TO) range; site codes are defined as in Fig. 1; points jittered for visual clarity.

To evaluate thermal tolerance across populations we used a two-step approach. First, we extracted LT_50_ estimates for each heat bar trial (‘trial’ = 18–30 snails from a population-acclimation treatment in the heat bar) using Firth’s bias-reduced logistic regression (Heinze and Schemper, 2002) due to complete separation of the survival data. Complete separation occurs when a predictor perfectly discriminates between binomial states. In our case, survival in each trial was consistent up to a certain temperature threshold after which all individuals died, and thus was completely separated (Fig. 4; Cheng et al. 2017). This lack of variation is problematic for traditional model estimation, thus necessitating the alternate approach. For these analyses we used the *brglm2* package in R (Kosmidis, 2021) to model the effect of final heat bar temperature on survival for each population and acclimation temperature treatment. Thus, each ‘run’ of the heat bar produced three LT_50_ measurements for the three ‘trials’ of population-acclimation treatments.

Second, as opposed to modelling LT_50_ as a function of population (e.g. using ANOVA), we used a regression-based approach using five environmental variables from each population to understand drivers of thermal tolerance over an environmental cline (Table S5). Once we extracted the LT_50_ from each trial, we then tested for geographic patterns in thermal tolerance by pairing each population’s environmental data (Table S5) with their extracted LT_50_ estimates. These environmental data were then used as a suite of predictors, in addition to the acclimation temperature of each trial, in a model-selection framework. We constructed generalized linear models with Gaussian error distributions using this set of environmental and acclimation predictors, and used small sample adjusted Akaike’s Information Criterion (AICc) to select models that had the greatest support against a null model. We chose our cut-off of well-supported models for model selection throughout as ΔAICc < 2 (Burnham and Anderson, 2002).

We further examined the difference between calculated thermal tolerances (LT_50_) and the habitat temperature of each population (hereafter referred to as WT; Deutsch *et al.*, 2008). We calculated WT as WT = LT_50_ − T_hab_, with T_hab_ as the maximum summer temperature. This method accounts for maximum water temperatures an organism could experience, which is likely to be a selective force across populations (Kingsolver *et al.*, 2013; Sunday *et al.*, 2014). We calculated separate WT estimates using LT_50_ values from the 20°C and 24°C acclimation temperatures to assess how thermal history may influence thermal sensitivity estimates. While we included the two Pacific sites in the data, we did not model an effect of invasion status because there was no overlap in T_hab_ values between oceans and due to limited population replication in the Pacific.

### Developmental traits

We used generalized linear mixed models to assess the fixed effects of acclimation temperature and environmental predictors and their interaction on developmental traits (hatching success, clutch size, development time). We included clutch as a random effect. For clutch size, we used a Conway–Maxwell Poisson error distribution because of initial overdispersion in the data (Chanialidis *et al.*, 2018). For hatching success of snails, we used a binomial error distribution with logit link function. For development time, we used a Gaussian distribution. For all development analyses, we used environmental predictors as defined in Table S5. For these analyses we used the *glmmTMB* package (Brooks *et al.*, 2017). We performed all thermal tolerance and developmental trait statistical analyses in R (v. 3.5.1, R Core Team, 2018).

## Results

### Thermal tolerance

**Figure 5:**
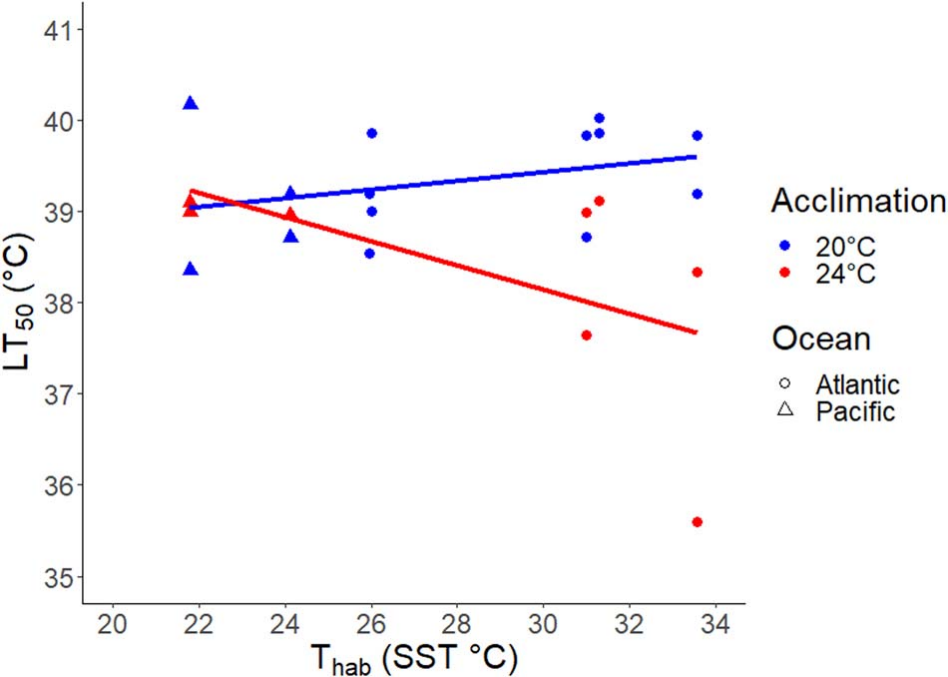
LT_50_ estimates of *Urosalpinx* hatchlings over their habitat maximum summer temperature and two experimental acclimation temperatures; T_hab_ is the maximum summer temperature.

The most supported model describing spatial patterns of thermal tolerance in *Urosalpinx* contained habitat temperature (T_hab_) as measured by the maximum summer habitat temperature at each site with an interactive effect with acclimation temperature (T_acc_). When acclimated at 20°C, thermal tolerance increased with habitat temperature significantly but with high variability (GLM, F_3,18_ = 4.51, *P* = 0.0417; Fig. 5; Table 1). When acclimated at 24°C, thermal tolerance decreased significantly with habitat temperature (GLM, F_3,18_ = 4.51, *P* = 0.0352; Fig. 5; Table 1). *Urosalpinx* acclimated at 20°C and 24°C had a cross-population mean thermal tolerance of 39.3 ± 0.61°C (*n* = 14) and 38.3 ± 1.22°C (*n* = 8; mean ± SD), respectively.

**Figure 6:**
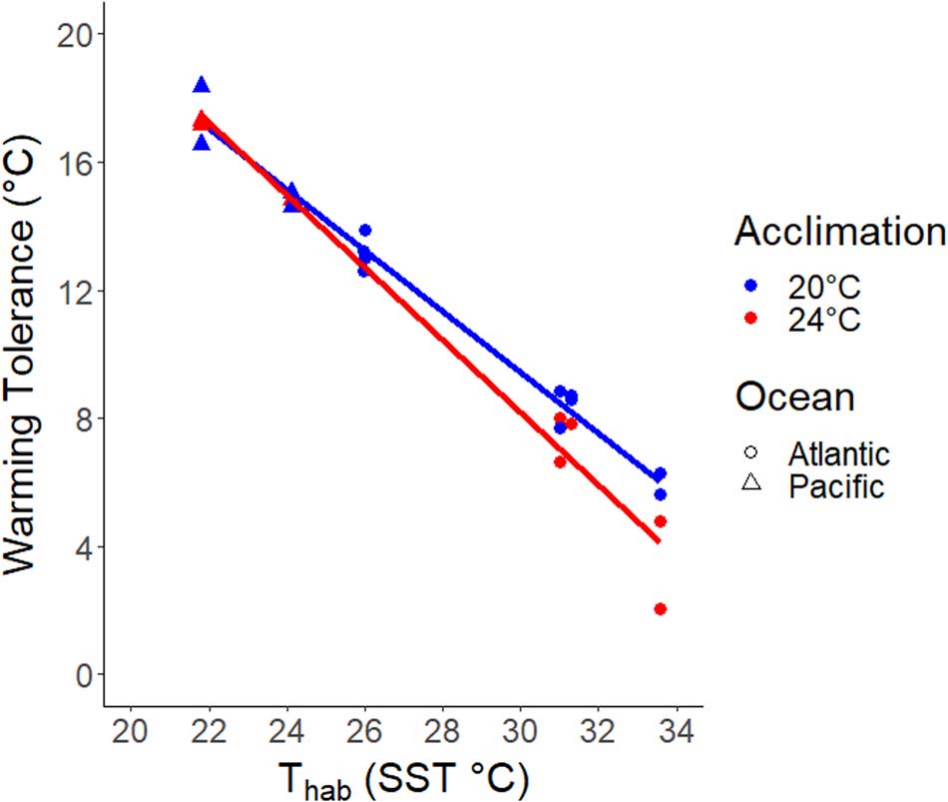
Latitudinal and oceanic trends in WT (LT_50_ − T_hab_), with T_hab_ being the maximum site summer temperature; trendline depicts the significant relationship between WT and T_hab_ at the 20°C and 24°C acclimations. Note that we include Pacific site data, but omitted invasion status as a predictor from analysis because of low sample size. T_hab_ is the maximum summer temperature.

**Table 1:** Parameter estimates for thermal tolerance, WT and developmental rate models

Parameter	Estimate	SE	t/z	*P*
Thermal tolerance R^2^ T_hab_*Acc (multiple/adjusted): 0.429/0.334				
Acc_20_ (Intercept)	17.2	12.4	1.39	0.182
Acc_24_	1.04	0.568	1.83	0.0838
T_hab_* Acc_20_	0.956	0.436	2.19	**0.0417**
T_hab_ * Acc_24_	−0.0454	0.0199	−2.28	**0.0352**
WT R^2^ T_hab_*Acc (multiple/adjusted): 0.975/0.971				
Acc_20_ (Intercept)	38.0	1.49	25.5	**<0.001**
Acc_24_	4.16	2.27	1.83	0.0838
T_hab_ * Acc_20_	−0.951	0.0533	−17.9	**<0.001**
T_hab_ * Acc_24_	−0.182	0.0797	−2.28	**0.0352**
Developmental rate R^2^_GLMM_ (marginal/conditional): 0.906/0.935				
Acc_20_ (Intercept)	46.143	1.756	26.283	**<0.001**
Acc_24_	−15.430	1.844	−8.368	**<0.001**
T_hab_ * Acc_20_	−0.463	0.105	−4.397	**<0.001**
T_hab_ * Acc_24_	0.286	0.111	2.577	**0.00996**

Values in boldface: significance levels of *P* < 0.05.

Multiple and adjusted R-squared values are presented for model-averaged and single-model GLMs.

For mixed-effect models (developmental rate), the marginal and conditional R-squared values are given, which estimate model explanatory power between fixed effects and fixed and random effects combined (Nakagawa and Schielzeth, 2013).

T_hab_, as determined via AICc model selection, is the maximum summer temperature for both thermal tolerance and WT and mean annual temperature for developmental rate.

### Warming tolerance

We found a strong pattern of decreasing WT (thermal tolerance − maximum habitat temperature) with increasing summer maximum site temperature for both acclimation temperatures (GLM F_3,18_ = 11.4; 20°C: *P* < 0.001, 24°C: *P* = 0.0352), but that WT was not significantly different between acclimations (GLM F_3,18_ = 11.4, *P* = 0.0838; Fig. 6; Table 1). Invasive pacific populations appeared to have the highest WT values, although we note that we did not explicitly model invasion status because of the low number of invasive population replicates (*n* = 2). The minimum calculated WT occurred in the Virginia population (‘Oyster’) at 24°C acclimation (2.03°C), while the largest WT occurred in the California (‘Humboldt’) population at 20°C acclimation (18.4°C).

### Developmental traits

**Figure 7:**
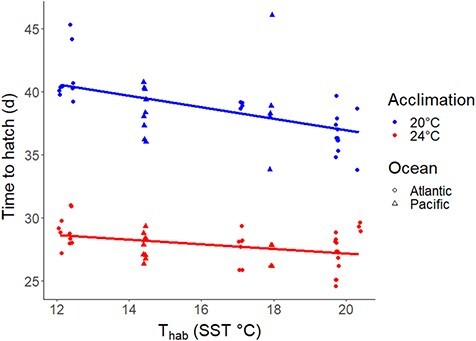
Developmental rate of *Urosalpinx* egg cases when acclimated at 20°C and 24°C; T_hab_ is the mean annual temperature; points jittered for visual clarity.

The hatching time of *Urosalpinx* egg cases decreased with greater mean annual habitat temperature of the source population (GLMM *n* = 39, *P* < 0.001) for egg cases reared at a common temperature of 20°C (Fig. 7; Table 1). At 20°C acclimation, the shortest developmental time occurred in egg cases from the southernmost Atlantic site [Folly Beach, 36.5 ± 3.53 days (SD)], while the greatest development time occurred in the northernmost Atlantic site (Great Bay, 41.8 ± 2.59 days). When acclimated at the higher temperature of 24°C, hatching time decreased across all sites (GLMM *n* = 46, *P* = 0.00996; Fig. 7; Table 1). The shortest development time at 24°C occurred in North Carolina (Beaufort; 26.8 ± 1.28 days), and despite the significant negative trend between habitat temperature and time to hatching, the slowest development rates occurred at both the northernmost and southernmost Atlantic sites (South Carolina: 29.3 ± 0.577 days; New Hampshire: 29.2 ± 1.47 days). Random effects of clutch gave intercept variance of 0.955 ± 0.977 (SD) and little difference between marginal (0.906; fixed effects only) and conditional (0.935; fixed and random effects) R^2^_GLMM_ (Nakagawa and Schielzeth, 2013). Multicollinearity was low (VIF < 2.5) for all well-supported developmental trait models. Both clutch size and hatching success metrics had multiple well-supported models, so we model averaged top models of clutch size and hatching success. None of the best-supported models were overdispersed (deviance < degrees of freedom).

Clutch size showed a significant but highly variable relationship with environmental predictor parameters (GLMM *n* = 85, *P* = 0.0086; maximum summer temperature; Table S6; Fig. S3), such that warm-origin populations had a larger number of embryos per egg case than their cold-origin counterparts. Hatching success increased with habitat temperature (GLMM *n* = 85, *P* = 0.0172; 75th percentile summer temperature), although there was considerable variation (Table S6; Fig. S3). Elevated acclimation temperature had no effect on hatching success (Table S6; Fig. S3). The random intercept of clutch for clutch size had a variance of 0.0557 ± 0.236 (SD), and each egg case’s hatching success had a variance of 0.373 ± 0.610 (SD). Taken together, the developmental metrics (particularly developmental rate and hatching success) indicate an increase in performance with increasing habitat temperature.

## Discussion

Thermal performance has often historically been assumed to be homogeneous within species, an assumption that can generate inaccurate forecasts of species response to climate change if there is adaptive differentiation across populations. There is increasing recognition that intraspecific variation may be common in the ocean (Kuo and Sanford, 2009; Zippay and Hofmann, 2010; Kelly *et al.*, 2012; Hong and Shurin, 2015; Pereira *et al.*, 2017; Sasaki and Dam, 2019). However, observations supporting this view are generally limited, particularly across populations of a species’ native and invasive ranges (but see Henkel *et al.*, 2009; Yu *et al.*, 2012; Hill *et al.*, 2013; Tepolt and Somero, 2014; Wesselmann *et al.*, 2020). Here, we found evidence for greater thermal tolerance in southern populations of oyster drills that experience higher habitat temperatures, in support of our hypothesis of local adaptation. However, when developmental acclimation temperature was increased, thermal tolerance decreased in southern populations (2.1%–6.4% decrease), contrary to expectations of greater thermal tolerance with higher acclimation. Further, we found diminished WTs of low-latitude Atlantic populations as compared to high-latitude Atlantic (native) and Pacific (invasive) populations, consistent with the non-compensating local adaptation model of WT (Fig. 1C). This follows our conceptual framework of how variation in thermal tolerance may be inverse to variation in WT (Fig. 1); thermal tolerance that compensates well with changes in the environment results in low variance in WT. In this study, we show that because thermal tolerance does not scale strongly with environmental temperature, variation in calculated WT is high between populations. These results suggest a striking contrast. Low-latitude native populations appear to exhibit high climate sensitivity and may become extirpated if warming continues ultimately resulting in range contraction. In contrast, the two Pacific (invasive) populations of *Urosalpinx* studied may be more likely to persist in a warming future because of a large buffer between current habitat temperatures and their thermal tolerance. *Urosalpinx* has well-documented impacts on native, foundational species such as Olympia oysters (*O. lurida*), and therefore will likely continue to drive cascading negative effects on native biodiversity into the future (Kimbro *et al.*, 2009; Cheng and Grosholz, 2016).

We found interactive effects of source population environment and acclimation temperature on thermal tolerance (Fig. 5). Populations reared at 20°C displayed a positive relationship between thermal tolerance and habitat temperature, consistent with other studies on marine invertebrates (Zippay and Hofmann, 2010; Sunday *et al.*, 2011; Kelly *et al.*, 2012; Pereira *et al.*, 2017; Sasaki and Dam, 2019). Although the differentiation in thermal tolerances appears small (~1.0°C across the range tested), this effect size is similar to other studies testing intraspecific variation in thermal tolerance (Fangue *et al.*, 2006; Kuo and Sanford, 2009; Jensen *et al.*, 2019). Interestingly, higher developmental acclimation temperature (24°C) resulted in a negative relationship between habitat temperature and thermal tolerance, or what we define as ‘negative plasticity’. At first glance, these results are counterintuitive given the tendency of higher acclimation to result in elevated thermal tolerances (Angilletta Jr., 2009; Pereira *et al.*, 2017; Sasaki and Dam, 2019). However, evidence of a negative response to higher acclimation temperature has been demonstrated in nudibranchs (Armstrong *et al.*, 2019) and salmonids (Blair and Glover, 2019; Del Rio *et al.*, 2019) in both developmental and within stage acclimations, albeit not between multiple populations. This negative plasticity in thermal tolerance from southern populations is suggestive of a tradeoff between elevated thermal tolerance and plasticity (Stillman, 2003; Armstrong *et al.*, 2019; van Heerwaarden and Kellermann, 2020). Southern populations have evolved elevated thermal tolerance in response to warm environmental conditions but have done so at the cost of plasticity extent. Because northern/invasive populations have lower evolved thermal tolerances, they do not exhibit such trade-offs with plasticity. It should be noted that our scope of inference is limited here because we were not able to quantify thermal tolerance of northern Atlantic sites at 24°C (Great Bay and Woods Hole) because we were not able to obtain enough juveniles from each treatment to run a heat bar trial. Furthermore, an acclimation of 24°C itself may be stressful for embryonic and newly hatched *Urosalpinx.* We originally chose 24°C as the higher acclimation temperature because it is below the measured thermal optima of juvenile, invasive range *Urosalpinx* (26.5°C, Cheng *et al.*, 2017), and hatchling survivorship, while invariant with acclimation temperatures in our study, has previously been shown to peak at 20°C and decrease at 25°C (Ganaros, 1958). However, because early life stages are often the most vulnerable to thermal stress, it is further possible that physiological stress is incurred in embryos and hatchlings at 24°C (Truebano *et al.*, 2018; Dahlke *et al.*, 2020; McKenzie *et al.*, 2020). Thermal stress can accumulate over time with heightened sublethal temperatures, resulting in reduced survivorship in what has been described as a tolerance landscape (Rezende *et al.*, 2020). These developmental acclimation effects are tested less often, but are important because ocean warming is occurring across seasonal cycles and can impact early development when many organisms are the most sensitive (Pechenik, 2006; Marshall and Morgan, 2011; Dahlke *et al.*, 2020). Our results point to the importance of carefully considering how seasonality of environmental exposure and ontogeny may affect thermal sensitivity across life stages. This is a critical consideration when designing experimentation tracking local adaptation across generations, especially with complex life stage organisms from environments with strong seasonal thermal fluctuations. Models that predict population persistence using adult thermal optima or tolerance may overpredict potential ranges by not considering heightened sensitivity of early life stages and the carry-over effects of warming during development.

Among environmental correlates, maximum habitat temperature best explained variation in thermal tolerance. Most studies use mean annual temperature in predicting variation in thermal tolerance, perhaps because these data are readily available and explain some variation in tolerance (e.g. Deutsch *et al.*, 2008; Hughes *et al.*, 2019). However, maximum habitat temperature is expected to be the main driver of thermal tolerance both within and across species (Hoffmann, 2010; Kelley, 2014; Pinsky *et al.*, 2019). Maximum temperatures should act as a ‘filtering’ agent such that a locally adapted population will have thermal tolerances selected for from standing genetic variation that allow it to persist in that environment (Bennett *et al.*, 2019; Pinsky *et al.*, 2019). Local thermal heterogeneity, driven by processes such as upwelling, tides and currents also mean that environmental metrics like latitude or mean temperature are not necessarily correlated with maximum habitat temperature (Baumann and Doherty, 2013). We found that maximum habitat temperature consistently drove variation in thermal tolerance spanning native and invasive ranges (Fig. 4). Our temperature records, obtained from buoys within 15 km and of similar habitat type of collection sites, offer a general view of the environmental conditions experienced by populations. However, given organismal body temperature itself may vary as a function of microhabitat and behaviour (Helmuth *et al.*, 2010), the exact maximum temperature each *Urosalpinx* population experiences may differ from those obtained via buoy data. As a result, we suggest future work consider testing relationships between upper thermal tolerance and maximum habitat temperatures along with mean temperature and/or latitude, as well as deploying collocated temperature loggers to refine these environmental parameters. By not directly correlating thermal tolerance with a major selective environmental force (i.e. maximum habitat temperature), patterns of local adaptation may be ignored or overstated, potentially wasting resources by managing populations that are not actually sensitive to climate change.

Diminished WT at warm-origin sites indicates that southern populations are closer to their thermal limit than their northern counterparts (Fig. 6) and that population origin has a stronger effect on climate sensitivity than does acclimation temperature. Interestingly, this result sets up a third potential pattern of thermal tolerance, habitat temperature and WT (see Fig. 1). Despite thermal tolerance being locally adapted, WT was not constant across populations, indicating that a third model of WT (what we call here ‘non-compensating’ local adaptation; Fig. 1C) between niche-conserved (Fig. 1A) and locally adapted populations (Fig. 1B) is possible. This is likely a result of thermal tolerance not being 1:1 correlated with decreasing habitat temperature. This decreasing relationship between WT and habitat temperature is consistent with studies that have examined intraspecific sensitivity to climate in crabs, nudibranchs and leaf miner moths (Gaitán-Espitia *et al.*, 2014; Pincebourde and Casas, 2015; Armstrong *et al.*, 2019), as well as studies of interspecific climate sensitivity (Deutsch *et al.*, 2008; Sunday *et al.*, 2011; Allen *et al.*, 2012; Diamond *et al.*, 2012; Vinagre *et al.*, 2016; Comte and Olden, 2017; Janion-Scheepers *et al.*, 2018). Taken together, this evidence supports the view that low-latitude populations appear to have high climate sensitivity (Tewksbury *et al.*, 2008; Pinsky *et al.*, 2019). In contrast, temperate populations have greater WT despite reduced thermal tolerance, perhaps because of exposure to lower environmental temperatures (Deutsch *et al.*, 2008; Janion-Scheepers *et al.*, 2018). Reduced WT at the warm edge of a population’s range also highlights the potential role of thermal tolerance in driving extirpation and range contractions at the trailing edge (Sunday *et al.*, 2012; Cahill *et al.*, 2014; Hardy *et al.*, 2014). Depending on the management goal for a species exhibiting this pattern of WT (control for *Urosalpinx*, conservation for others), this potential for local extinction and species range contraction at the warm trailing edge is of critical interest and may call for resource reallocation away from warm, trailing-edge populations.

We found strong evidence for faster developmental rates for populations sourced from warm habitats and higher developmental acclimation at 24°C resulting in overall faster growth than at 20°C (Fig. 7). Warm, southern populations developed the fastest at all acclimation temperatures, as expected by biogeographic theory of embryonic development rate in marine ectotherms (Lonsdale and Levinton, 1985; Collin, 2003; Weydmann *et al.*, 2015). Increased development rate at lower latitudes may result from simple increases in metabolic rate with habitat temperature (Lonsdale and Levinton, 1985), or potentially because of selection arising from heightened risk of predation in tropical low-latitude systems (Schemske *et al.*, 2009). Interestingly, the fastest development rate occurs at the acclimation temperature (24°C) and populations (low-latitude Atlantic) that had the lowest thermal tolerance, suggesting potential trade-offs across life stages (Stillman, 2003). While both were highly variable, hatching success increased with habitat temperature, such that warm populations develop faster and have higher survivorship, and clutch size decreases with higher habitat temperature. Therefore, warm-origin populations spawn smaller egg case clutches, which develop quicker, and have a greater chance of developing successfully. As juveniles, these warm-origin populations show higher thermal tolerance (Fig. 5), but only at a lower acclimation temperature. Additionally, the reduced number of embryos per egg case (low ‘embryo packing’) in warm populations may be a product of a tradeoff between embryo density and oxygen availability within each egg case in warm waters (Lee and Strathmann, 1998; Fernández *et al.*, 2007). These results indicate the potential for rapid embryonic development to result in trait performance trade-offs in later life stages as a result from increased metabolic demand during embryonic growth (Have, 2002; Pörtner *et al.*, 2006; Del Rio *et al.*, 2019). If development rate is maximized at each acclimation temperature, then enzymatic activity may itself be maximally efficient at these temperatures, and thermal tolerance is reduced due to inefficient enzymatic reactions at elevated temperatures (Somero, 1995; Have, 2002). One caveat of our findings is that we quantified embryonic performance in July in order to synchronize experimental treatments. It is possible that the variation in hatching success and clutch size may reflect phenological shifts in spawning seasons (Carriker 1955). Future efforts should quantify these aspects of spawning and development across the spawning seasons in order to fully resolve the potential range of intraspecific variation and plasticity in *Urosalpinx*. Our results point to the mechanistic importance of early life stage experiences on trait performance and tradeoffs in subsequent life stages and the need for future research to characterize trait performance and optima across life stages (Pechenik, 2006; Slotsbo *et al.*, 2016).

We found invasive and high-latitude native *Urosalpinx* populations to be the least sensitive to climate impacts based on their high WT values, suggesting that these populations will persist in their environments. We acknowledge that sampling in the invasive region was limited to two populations and that current data is unable to determine whether greater WT of these populations is due to population genetics (e.g. founder effect or population bottleneck from introduction) or due to the environment alone (i.e. a large buffer from current habitat temperatures and thermal tolerance). Current efforts are underway to resolve genetic differences among populations in the native and invasive range. Nonetheless, the high WT observed at the California sites is a concern for native biodiversity because near term warming is likely to increase the predatory impact of *Urosalpinx* on native species, including consumption of Olympia oysters (*O. lurida*) that are the focus of conservation and restoration efforts (Cheng *et al.*, 2017). Further, heightened development rate at greater acclimation suggests that embryos will develop faster with potentially higher metabolic rates, increasing the consumption of newly hatched juveniles on oysters. From a community ecology perspective, these differing climate sensitivities between *Urosalpinx* throughout native and invasive ranges demonstrates the potential for indirect impacts of climate change on native biodiversity. Interactions between *Urosalpinx*, climate and humans highlights ‘trophic skew’, the reorganization of biological communities with species loss from extinction and species gain from invasion (Grosholz, 2002; Duffy, 2003; Byrnes *et al.*, 2007). As marine environments warm, native species will experience both abiotic pressure from warming as well as pressure from the persistence and proliferation of invasive, warm-origin predators like *Urosalpinx* (Cheng and Grosholz, 2016). Early eradication and control of these resilient invasive predators may assist native species by removing a biotic pressure as natives adapt or migrate in the face of climate change, thereby potentially reducing of trophic skewness (Byrnes *et al.*, 2007; Grosholz and Ruiz, 2009; Cheng *et al.*, 2017).

In conclusion, our work demonstrates the importance of taking an intraspecific approach to examining thermal performance and sensitivity to climate. Such variation can have large implications for forecasts of species responses to climate change that often assume homogeneity across populations, thereby missing the possibility of more resilient populations under climate change. We found largely negative effects of developmental acclimation on thermal tolerance, a crucial consideration given that climate change occurs across temporal scales (e.g. seasons) and will result in biological effects both within and across life stages. We also show that integrating environmental data can provide a more complete picture of population-level sensitivity that may drive geographic range contractions. Taken together, this approach can be useful for developing an understanding of climate impacts on populations across their native and invasive ranges. Such a perspective is useful for clarifying potential interactions between climate and biological invasions that can erode native biodiversity. The variation in thermal physiology we demonstrate here supports the necessity of using data from multiple populations when making ecological forecasts of climate change.

## Funding

This work was supported by the PADI Foundation (40638 to A.R.V.), the American Malacological Society Melbourne R. Carriker Student Research Award to A.R.V., the National Science Foundation (BIO-OCE 2023571 to B.S.C. and L.M.K), the National Institute of Food and Agriculture, U.S. Department of Agriculture, the Center for Agriculture, Food and the Environment and the Department of Environmental Conservation at the University of Massachusetts Amherst (under project number MAS00558). The contents are solely the responsibility of the authors and do not necessarily represent the official views of the USDA or NIFA.

## Supplementary Material

Villeneuve_lt50_MS_supplementary_4_15_coab039
