# Supplementary material for: Diminished warming tolerance and plasticity in low-latitude populations of a marine gastropod
Source: Conserv Physiol. 2021 Jun 11;9(1):coab039. doi: 10.1093/conphys/coab039 (PMC8201192; doi:10.1093/conphys/coab039)
Supplement: Villeneuve_lt50_MS_supplementary_4_15_coab039 [file villeneuve_lt50_ms_supplementary_4_15_coab039.docx]

**Table S1.** Collection sites and environmental data sources of broodstock *Urosalpinx cinerea* used in experimentation.

| Site | Code | State | Temperature Data Source | Year | Data Lat | Data Lon | Collection Lat | Collection Lon | Data Dist. from site (km) |
| --- | --- | --- | --- | --- | --- | --- | --- | --- | --- |
| Great Bay | GB | NH | Jackson Estuarine Lab (University of New Hampshire) and NERR (station grbulbwq) | 2018 | 43.107 | -70.863 | 43.089 | -70.866 | 1.94 |
| Woods Hole | WH | MA | NOAA NDBC (station BZBM3) | 2018 | 41.524 | -70.671 | 41.577 | -70.641 | 6.41 |
| Oyster | OY | VA | Virginia Coast Reserve LTER (station OYST; (Porter *et al.*, 2019) | 2018 | 37.289 | -75.923 | 37.288 | -75.924 | 0.02 |
| Beaufort | BF | NC | NOAA NDBC (station BFTN7) | 2018 | 34.717 | -76.671 | 34.718 | -76.671 | 0.1 |
| Folly Beach | FB | SC | NOAA NDBC (station CHTS1) | 2018 | 32.781 | -79.924 | 32.660 | -79.943 | 13.47 |
| Humboldt | HM | CA | CeNCOOS/Wiyot Tribe (station Indian Island) | 2015 | 40.815 | -124.158 | 40.85 | -124.085 | 7.27 |
| Tomales | TO | CA | Grozholz and Largier Labs; Bodega Ocean Observation Node (BOON), Bodega Marine Laboratory, University of California, Davis (Hollarsmith *et al.*, 2020) | 2015 | 38.118 | -122.867 | 38.128 | -122.864 | 1.15 |


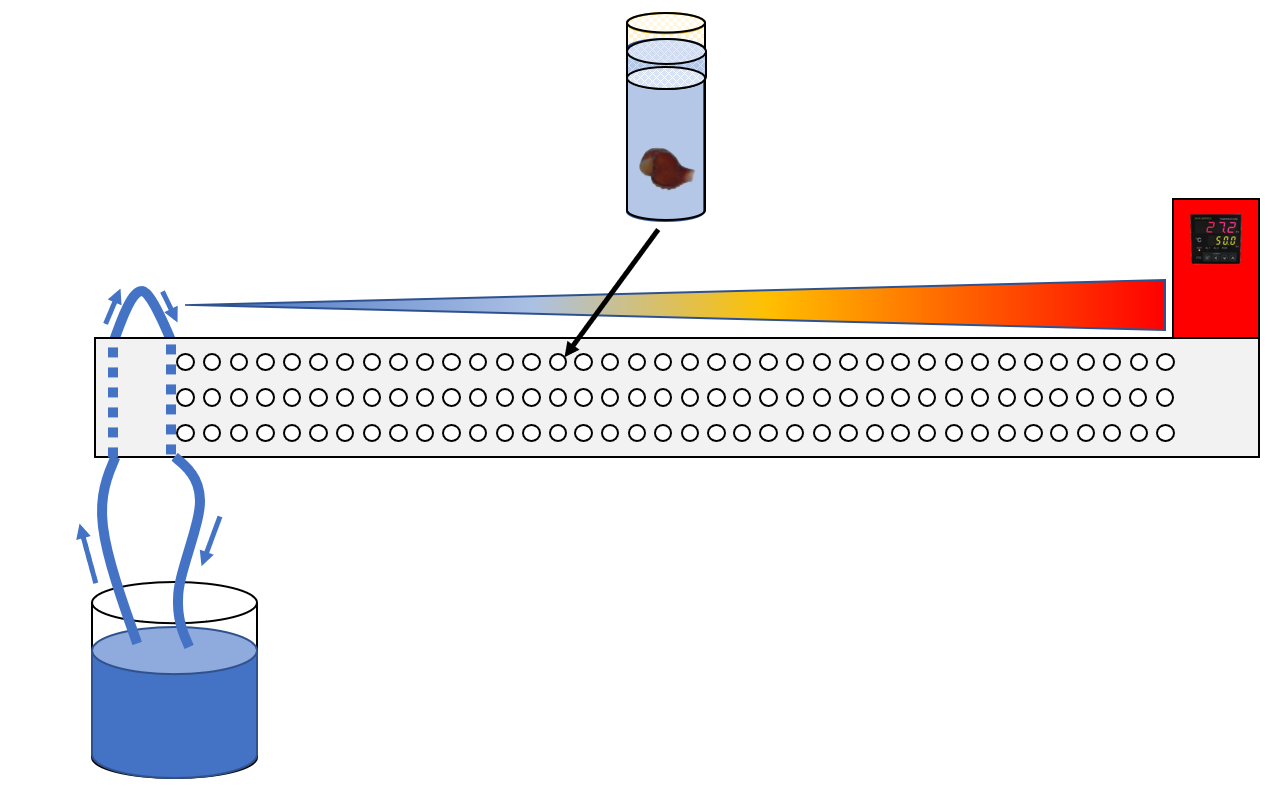


**Fig. S1.** Illustration of experimental heat bar array to test LT_50_ in *Urosalpinx cinerea.*

**Table S2**. The interactive effects of scaled and centered weight and final heat bar temperature on survival, modeled using binomial bias-reduced generalized linear models and the *logistf* package (Heinze *et al.*, 2020), as binomial data was completely separated. Significant *P* values are bolded.

| Parameter | Estimate | SE | Chi-square | *P* |
| --- | --- | --- | --- | --- |
| (Intercept) | 3.1 | 0.421 | inf | **> 0.0001** |
| Final temperature (scaled, centered) | -8.28 | 0.987 | inf | **> 0.0001** |
| Weight (scaled, centered) | -0.289 | 0.198 | 2.67 | 0.258 |
| Final temperature * weight (scaled, centered) | 1.2 | 0.383 | 2.84 | 0.092 |

**Table S3.** Ramping setpoints used in Prot_1_ and Prot_2_ heat bar experiments. Time reflects the timepoint within the context of a 5 hour heat bar experiment.

| **Time (hrs)** | **Setpoints (°C)** | **Ice in** |
| --- | --- | --- |
| 0 | ambient | Yes |
| 0.5 | 25 | No |
| 1 | 30 | Yes |
| 1.5 | 35 | No |
| 2 | 40 | Yes |
| 2.5 | 45 | No |
| 3 | 50 | No |
| 3.5 | 55 | No |
| 4 | 60 | No |
| 4.5 | 60 | No |
| 5 | end | end |


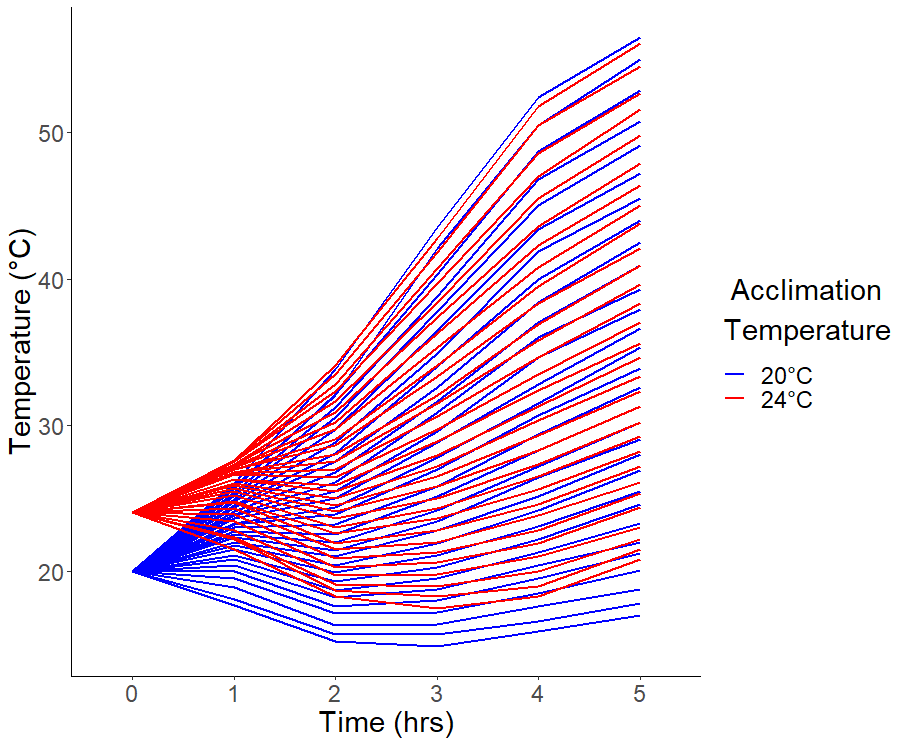


**Figure S2.** Each line represents the temperature trajectory through time of a single horizontal position in the heat bar. Ramps are from two selected heat bar runs on 21/8/19, 28/8/19.

**Table S4.** Summary of heat bar run data, indicating the total number of extracted data points for each trial run.

| **Site** | **Acclimation** | **Number of Runs** | ***N* (individual snails)** |
| --- | --- | --- | --- |
| FB | 20 | 2 | 60 |
| FB | 24 | 1 | 30 |
| BF | 20 | 2 | 60 |
| BF | 24 | 2 | 60 |
| OY | 20 | 2 | 60 |
| OY | 24 | 2 | 60 |
| WH | 20 | 2 | 60 |
| WH | 24 | 0 | 0 |
| GB | 20 | 2 | 60 |
| GB | 24 | 0 | 0 |
| TO | 20 | 2 | 58 |
| TO | 24 | 1 | 30 |
| HM | 20 | 2 | 60 |
| HM | 24 | 2 | 54 |
|  | Total | 22 | 652 |

**Table S5.** Site environmental metrics used as predictors.

| Site | Latitude | AnnualMean (°C) | Summer Mean (°C) | 75th Percentile, Summer (°C) | 90th Percentile, Summer (°C) | Maximum Summer (°C) |
| --- | --- | --- | --- | --- | --- | --- |
| Great Bay, NH | 43.107 | 12.40 | 20.89 | 22.91 | 23.70 | 25.96 |
| Woods Hole, MA | 41.524 | 12.10 | 21.35 | 23.60 | 24.30 | 26.00 |
| Oyster, VA | 37.289 | 17.08 | 27.54 | 28.92 | 30.07 | 33.57 |
| Beaufort, NC | 34.717 | 19.74 | 28.28 | 29.10 | 29.80 | 31.00 |
| Folly Beach, SC | 32.781 | 20.35 | 29.12 | 29.80 | 30.00 | 31.30 |
| Humboldt, CA | 40.815 | 14.43 | 17.36 | 18.77 | 19.78 | 21.80 |
| Tomales, CA | 38.128 | 17.91 | 20.92 | 22.15 | 22.71 | 24.12 |


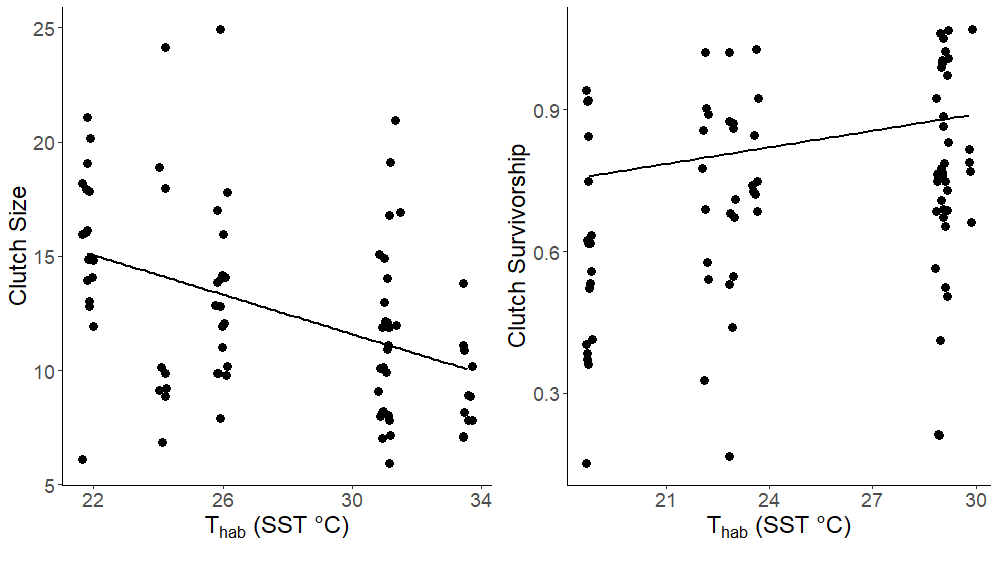


**Figure S3.** Clutch size (number of capsules) and hatching success (proportion successfully developed embryos) over summer maximum temperatures and 75^th^ percentile SST of summer months, respectively. Points are jittered for clarity. Regression lines represent a best-fit model of the data.

**Table S6.** Conditional model-averaged coefficients of *Urosalpinx* hatchling clutch size and hatching success. All models fell below cutoff of ΔAIC < 2. R^2^_GLMM_ , or marginal/conditional R^2^ , estimates model explanatory power of the fixed effects and fixed and conditional effects combined (fixed/fixed + conditional; Nakagawa & Schielzeth, 2013).

| Parameter | Estimate | SE | Adjusted SE | z | *P* | *R^2^_GLMM_* |
| --- | --- | --- | --- | --- | --- | --- |
| **Clutch Size** |  |  |  |  |  |  |
|  |  |  |  |  |  |  |
| (Intercept) | 3.30 | 0.337 | 0.342 | 9.656 | **<0.0001** | - |
| Maximum summer | -0.0326 | 0.0122 | 0.0123 | 2.63 | **0.0086** | 0.059/0.295 |
| 90th percentile summer | -0.0319 | 0.0125 | 0.0127 | 2.51 | **0.0121** | 0.062/0.294 |
| 75th percentile summer | -0.0300 | 0.0125 | 0.0127 | 2.37 | **0.0177** | 0.068/0.294 |
| Summer mean | -0.0272 | 0.0118 | 0.0120 | 2.27 | **0.0229** | 0.072/0.292 |
| **Hatching Success** |  |  |  |  |  |  |
| (Intercept) | -0.407 | 1.77 | 1.78 | 0.229 | 0.819 | - |
| 75th percentile summer | 0.0786 | 0.0325 | 0.0330 | 2.38 | **0.0172** | 0.0130/0.0542 |
| Summer mean | 0.0732 | 0.0307 | 0.0311 | 2.35 | **0.0186** | 0.128/0.543 |
| 90th percentile summer | 0.0773 | 0.0334 | 0.0339 | 2.28 | **0.0224** | 0.121/0.5432 |
| *Acc_24_ | -0.118 | 0.1460 | 0.148 | 0.799 | 0.424 | - |
| Latitude | -0.0864 | 0.0436 | 0.0442 | 1.95 | 0.0507 | 0.0980/0.543 |
| Maximum | 0.0650 | 0.0339 | 0.0344 | 1.89 | 0.0590 | 0.0879/0.543 |

**Citations**

Heinze, Ploner M, Jiricka L (2020) Firth’s Bias-Reduced Logistic Regression.

Hollarsmith JA, Sadowski JS, Picard MMM, Cheng B, Farlin J, Russell A, Grosholz ED (2020) Effects of seasonal upwelling and runoff on water chemistry and growth and survival of native and commercial oysters. *Limnology and Oceanography* 65: 224–235.

Nakagawa S, Schielzeth H (2013) A general and simple method for obtaining R^2^ from generalized linear mixed-effects models. *Methods in Ecology and Evolution* 4: 133–142.

Porter J, Krovetz D, Spitler J, Williams T, Overman K, Nuttle W (2019) High and Low Tides of Hog Island Bay, Redbank, VA, and Oyster, VA for the Virginia Coast Reserve 2007-2018.
